# Evaluation of 5 µm Superficially Porous Particles for Capillary and Microfluidic LC Columns

**DOI:** 10.3390/chromatography2030502

**Published:** 2015-08-04

**Authors:** James P. Grinias, Robert T. Kennedy

**Affiliations:** 1 Department of Chemistry, University of Michigan, Ann Arbor, MI 48109, USA; 2 Department of Pharmacology, University of Michigan, Ann Arbor, MI 48109, USA

**Keywords:** liquid chromatography, superficially porous particles, core-shell, microfluidics, chip-LC, capillary LC

## Abstract

Large-size (4–5 µm) superficially porous particles yield lower plate heights (e.g., the minimal reduced plate height or *h_min_* ≈ 1.5) than fully porous particles of a similar size when packed into large-bore columns. This property allows for better chromatographic performance without the higher pressures required for smaller particles. This study explores the use of such particles in microfluidic LC columns where materials and fitting pressure limits can constrain the size of particle used. The theoretically predicted performance improvements compared to fully porous particles were not demonstrated in capillary columns (with *h_min_* ≈ 2 for both particle types), in agreement with previous studies that examined smaller superficially porous particles. Microfluidic columns were then compared to capillary columns. Capillary columns significantly outperformed microfluidic columns due to imperfections imposed by microfluidic channel asymmetry and world-to-chip connection at the optimal flow rate; however, superficially porous particles packed in microfluidic LC columns had flatter plate height versus flow rate curves indicating potential for better performance at high reduced velocities.

## 1. Introduction

Since the introduction of a new generation of superficially porous particle (SPP) technology for LC columns in 2006 [1, 2], the popularity of these particles as a stationary phase support has grown tremendously [3–9] and is expected to continue increasing [10–12]. Their popularity results from improved efficiency compared to fully porous particles (FPPs) due to reduced stagnant mobile phase effects and eddy dispersion (resulting from a more homogenous bed structure) [3]. As a result, SPPs can have better efficiency than comparably sized FPPs or equivalent performance to smaller FPPs but at a lower pressure requirement. Columns packed with SPPs of 2.7 µm in diameter (*d_p_*) became widely used because their chromatographic efficiency matched sub-2-µm FPPs without requiring more expensive ultra-high pressure pumps [3]. Since those original 2.7 µm SPPs were released [1, 2], a wider range of sizes has become available [13]. The use of sub-2-µm SPPs has focused on high speed, high efficiency separations, but require UHPLC instruments with 1,000 bar (or higher) pressure limits [14–16]. Newer 4–5-µm SPPs have been used to increase speed and/or efficiency compared to FPPs [17–20] when using standard HPLC instruments where operating pressure is typically limited to 400 bar [17, 21].

Although SPPs have become prevalent in columns with inner diameters from 2.1–4.6 mm, only a few reports of their use in capillary LC formats have been described [22–28]. Interestingly, the performance improvement of SPPs compared to FPPs is not as significant in capillaries as it is for the larger bore columns described above. This difference may be due to beds packed with SPPs having “wall regions”, *i.e.*, structures disturbed by the column wall, that exist further from towards the bulk packing in the radial direction than FPPs do [24, 26]. This increases bed heterogeneity more in a capillary than it would in a larger diameter column (where wall effects have less impact on efficiency), thus eliminating expected efficiency gains observed in those types of columns. These studies have employed sub-3 µm SPPs, so the use of large-size SPPs for improved performance compared to FPPs for capillary LC remains to be explored.

SPPs have also not been widely used in microfluidic LC [29–32]; however, their use in chips is attractive because a current limitation of chips is the difficulty of implementing materials and connections suitable for high pressure [29, 30]. Therefore, SPPs that can achieve higher plate counts with larger sizes (and thus, lower flow resistances) represent an interesting, yet unexplored, opportunity to improve performance for chips. To that end, this study evaluates the efficiency and flow resistance of microfluidic LC columns packed with ~5 µm SPPs. This performance is compared to FPPs, specifically for small molecule separations, to determine which option could be more useful in chip LC where pressure is limited. Further, we compare the performance of chips and capillaries packed with the same particles to determine the effect of column geometry on the performance for this type of support.

## 2. Experimental Section

### 2.1. Chemicals and Materials

All reagents were purchased from Sigma-Aldrich (St. Louis, MO, USA) with the following exceptions. HPLC grade acetonitrile (MeCN) was from Burdick and Jackson (Honeywell, Muskegon, MI, USA). Lumiflavin was purchased from Santa Cruz Biotechnology, Inc. (Dallas, TX, USA) and a sample of potassium silicate for Kasil frits was provided by PQ Corporation (Valley Forge, PA, USA). Twenty-five and 75 µm inner diameter fused silica capillary was purchased from Polymicro Technologies (Phoenix, AZ, USA). Five-micrometer Alltima fully porous C18 particles were from Grace Davison (Deerfield, IL, USA). Five-micrometer Raptor ARC C18 superficially porous particles were generously donated by Restek Corporation (Bellefonte, PA, USA). Particle sizes were characterized using a Zeiss 1455VP Scanning Electron Microscope (SEM) (Jena, Germany) for imaging and ImageJ software (NIH, Bethesda, MD, USA) for analysis (*n* ≈ 100 particles measured).

### 2.2. Glass Chip Fabrication

Glass chips were fabricated using standard photolithography and wet etching techniques [33–37]. The channel for the chromatographic bed was 50 µm deep, 110 µm wide (full channel width) and 6 cm long (dimensions were selected to mimic the channel area of a 75 µm i.d. capillary). During channel etching, other sections of the chip were covered with HF-resistant tape (Semiconductor Equipment Corporation, Moorpark, CA, USA). A small gap was placed in the original photomask at 5.5 cm so that a small weir (~8 µm deep) would form during etching in order to retain particles during packing by the keystone effect [38–40]. After etching, access holes were drilled all the way through the substrate with a #92 (200 µm) drill bit (Kyocera Precision Tools, Inc., Costa Mesa, CA, USA) using a computer numerical control (CNC) machine (Cameron Micro Drill Press, Sonora, CA, USA), followed by a second counterbore hole halfway through the substrate with a #79 (368 µm) drill bit. The glass slides were then washed for 20 minutes in piranha solution (4:1 H_2_SO_4_:H_2_O_2_) and for 40 min in heated RCA solution (1:1:5 NH_4_OH:H_2_O_2_:H_2_O). Slides were rinsed with water, covered with a second slide of equal dimensions (but without any etching), and annealed at 610 °C for 8 h.

### 2.3. Column Preparation

A C-clamp fitting used for connecting capillaries to glass chips using a PEEK nut (IDEX Health & Science, Oak Harbor, WA, USA) and a PTFE ferrule (Chromatography Research Supplies, Inc., Louisville, KY, USA) was previously described for on-chip column packing in [39]. Such a fitting was used to couple the chip to a gas pressure packing vessel to deliver particles into the column channel with a 50 µm i.d. capillary. A vial containing a 20 mg/mL slurry of a given particle type in acetone was sonicated for 10 minutes and then placed into the packing vessel with the bottom of the capillary placed into the slurry. The pressure was increased to 60 bar (under the fitting pressure limit) until the column channel (5.5 cm) was completely filled with particles at which point the pressure was slowly released. The slurry was replaced with 50:50 H_2_O:MeCN, the pressure was again increased to 60 bar for 20 min to flush out any residual acetone, and the pressure was slowly released one final time prior to column characterization.

Capillary columns were packed in a method similar to previously reported techniques [41, 42] with some differences briefly described here. A 300 µm window for laser induced fluorescence (LIF) detection ~1 mm from the end of a 75 µm i.d. capillary was created using an electric arc. Outlet frits were then formed in the end of the capillaries using the Kasil method [43] where the tubing is pushed onto a glass microfiber filter (Whatman, GE Healthcare Life Sciences, Pittsburgh, PA, USA) wetted with a 1:1 (v:v) ratio of potassium silicate and formamide and then dried for at least two hours at 70 °C. These column blanks were then placed directly into the slurry vial in the packing vessel and a similar packing protocol to that used for the on-chip columns was used. The column blanks were then cut to length (6 cm) before characterization. For both capillary and microfluidic formats, three columns of each particle type were packed for comparisons (12 columns total).

### 2.4. Column Characterization and Analysis

**Figure 1:**
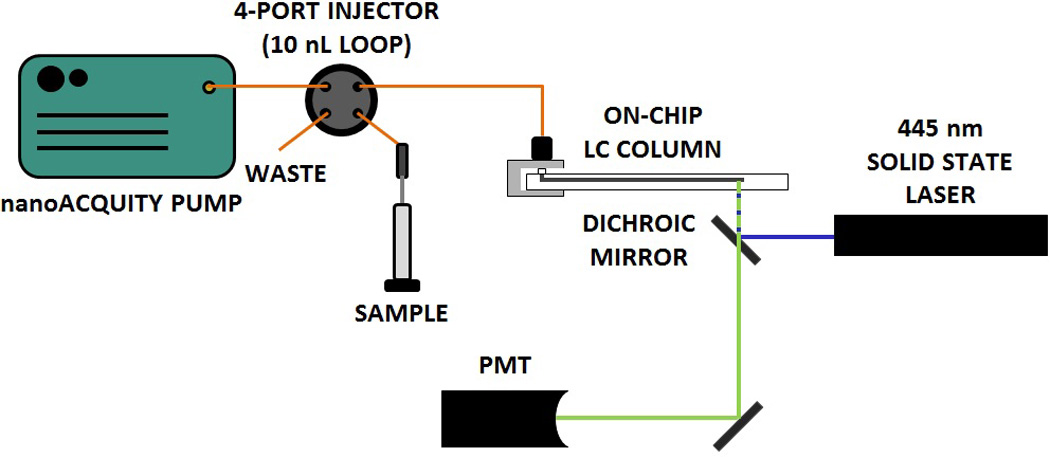
Instrument for efficiency measurements of chip-LC columns. The orange lines indicate capillary connections, the blue lines indicate excitation light (440 nm), and the green lines indicate emission light (490 nm). A C-clamp fitting [39] is used to connect capillaries to the on-chip channel.

Mobile phase was delivered to the columns using a nanoAcquity Binary Solvent Manager pump (Waters Corp., Milford, MA, USA). The pump was connected to a four-port, 10 nL internal loop injector (VICI, Houston, TX, USA) used for 100 ms actuated time-gated injections [44]. For chip columns, the injector was connected to the chip using the fitting described above with a 16 cm, 25 µm i.d. connecting tubing between the two. For capillary columns, an identical length of connecting tubing was used, but was connected to the column using a PicoClear fitting graciously provided by New Objective, Inc. (Woburn, MA, USA). On-column detection was achieved using laser-induced fluorescence with the laser focused at a point 1 mm before the outlet frit (Figure 1). The source consisted of a 440-nm, 15-mW solid-state laser (CrystaLaser, Reno, NV, USA) with a 436 ± 10 nm bandpass filter and 460 nm longpass dichroic mirror prior to excitation. Emission was filtered using a 490 ± 10 nm bandpass filter and then detected by a photomultiplier tube (R1477, Hamamatsu, Bridgewater, NJ, USA). Current from the PMT was amplified and filtered (10 Hz lowpass) by a Stanford current preamplifier (Sunnyvale, CA, USA) and acquired using an in-house written LabView (National Instruments, Austin, TX) program at 20 Hz. Retention times and peak variances were determined using an iterative statistical moments algorithm (±3σ integration limits) [45] in Igor Pro 6.0 (Wavemetrics, Inc., Lake Oswego, OR, USA). Further data analysis was conducted in both Igor Pro and Excel 2007 (Microsoft, Redmond, WA, USA).

To determine column performance, plate counts of lumiflavin were measured at a number of flow rates between 25 and 600 nL/min with 70:30 H_2_O:MeCN (+0.1% trifluoroacetic acid) used as the mobile phase. Mobile phase velocities were calculated using the elution times of a dead-time marker (riboflavin). To accurately evaluate column performance, the retention time was corrected for the delay of traveling from the injection valve to the column inlet (Figure 1). Corrections for extra-column band broadening associated with this transit were made by measuring a lumiflavin peak in 25 µm i.d. connecting tubing at a position equal to where the column would be connected (16 cm) [46]. Briefly, the variance for this transfer was measured and then subtracted from the variance measured on column. Further information on these corrections can be found in the Supporting Information. For a given instrument flow rate, the corrected plate counts and mobile phase velocities for three columns (with the same column format and particle type) were averaged to calculate reduced plate height-velocity (*h*-*v*) curves [47]. Diffusion coefficients for these calculations were estimated using the Wilke-Chang Equation [48].

## 3. Results and Discussion

### 3.1. Preparation and Use of Chip-LC Columns

**Figure 2:**
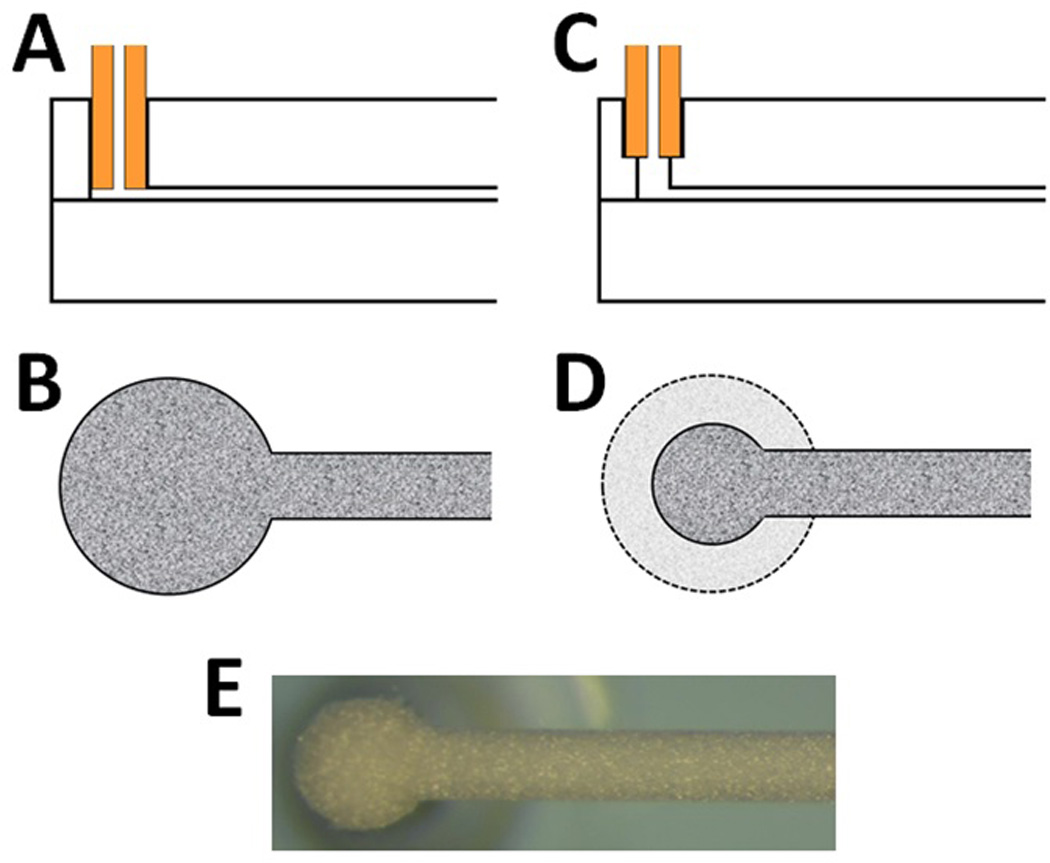
(**A**) Diagram indicating capillary placement into the chip with access hole directly drilled using a 368 µm drill bit. (**B**) Column inlet with access hole directly drilled using a 368 µm drill bit. (**C**) Diagram indicating capillary placement into the chip with counterbore access hole directly drilled using 368 µm and 200 µm drill bits. (**D**) Column inlet with counterbore access hole directly drilled using 368 µm and 200 µm drill bits (dotted region indicates column inlet with just a 368 µm drill bit used). (**E**) Optical microscope image of packed column inlet with counterbore access hole directly drilled using 368 µm and 200 µm drill bits.

To evaluate on-chip columns effectively, it was necessary to eliminate extra-column effects as much as possible. Initially, capillary access holes (368 µm in diameter) to the column were drilled all the way through the glass slide for connection by the C-clamp fitting (Figure 2A,B). However, reproducibly connecting new capillary (when switching from larger i.d. tubing for packing to smaller i.d. tubing for column analysis) directly at the head of the column was difficult due to capillary movement in the axial direction as the nut in the C-clamp fitting was tightened. Misaligning the capillary in this manner either disrupted the packed bed structure (if pushed too far) or left large dead volumes at the inlet that greatly reduced column efficiency (if not pushed far enough). To eliminate this effect, a counterbore hole design was used so that the capillary would rest against a secondary surface during tightening and not lower into the column (thus disturbing the packed bed structure) (Figure 2C). A second advantage of this method was that the diameter of the expansion at the end of the column channel (created from the drill bit) was nearly cut in half (down to 200 µm), reducing broadening in this region of the column (Figure 2B,D). Because it is difficult to successfully align these drill bits and the channel visually, a CNC machine was utilized to ensure correct access hole placement. Using CNC for alignment had the added benefit of ensuring that these holes were placed directly at the end of the channel and centered, preventing undesirable broadening zones that occur when the holes are not placed in this position (a common occurrence with visual alignment). With this technique, only a small dead volume between the connecting tubing and the head of the column exists due to the shape of the drill bit [49]. In the future, drill bits even smaller than 200 µm could be used for the center access hole (though bit fragility increases with decreasing diameter) or flat-tipped bits could be used [49] to reduce dead volumes in this type of connection.

### 3.2. Column Performance in Chips and Capillaries

The performance of both FPPs and SPPs in chip and capillary formats were evaluated using *h*-*v* plots for the test analyte lumiflavin (with riboflavin acting as an unretained dead time marker). For chip-LC columns, the minimum reduced plate height for the FPPs (*h_min_* = 4.9) was slightly better than that of the SPPs (*h_min_* = 5.6). However, the slope of the *h*-*v* curve was noticeably steeper for the FPPs (reduced *c*-terms of .20 compared to 0.09 for SPPs). When switching to capillary columns, the *h* greatly improves and the performance difference between the two particle types is very small (*h_min_* = 1.8 for FPPs and *h_min_* = 1.9 for SPPs). If the smaller diameter for the SPPs (as measured by SEM) is accounted for (using non-reduced terms), the plate counts at *H_min_* are nearly identical for the two particle types when packed into chips and about 10% higher for the SPPs in capillary columns (Table 1).

**Figure 3:**
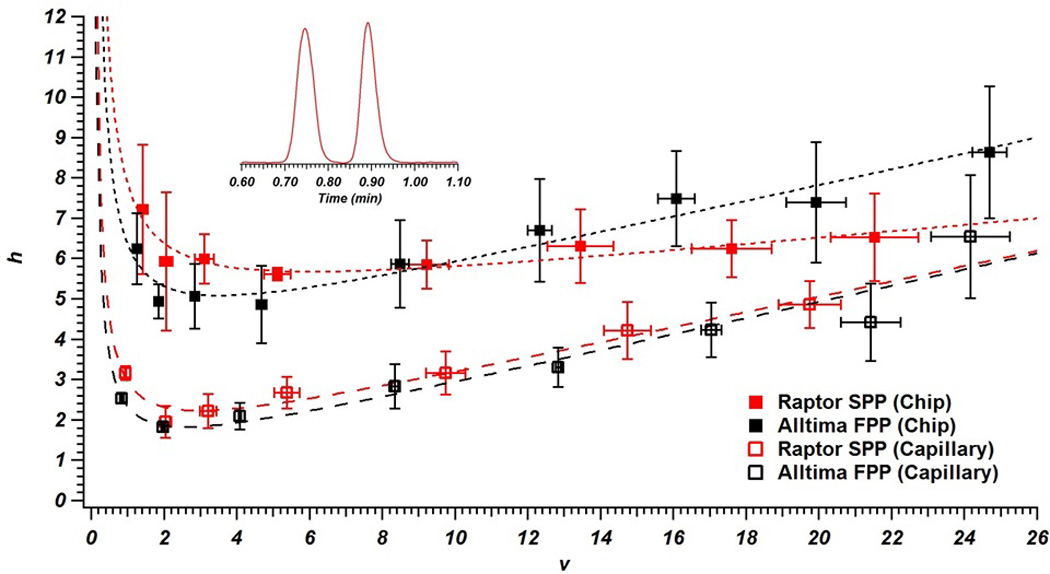
Reduced *h*-*v* curves for lumiflavin on columns packed with Raptor 5 µm superficially porous particles (*k'* ≈ 0.2) and Alltima 5 µm fully porous particles (*k'* ≈ 0.3) into capillaries (column length = 5.9 cm) and microfluidic chips (column length = 5.4 cm). Three columns of each type were packed and error bars reflect ±1 standard deviation (each data point corresponds to an equal pump flow rate). Data was corrected for extra-column band broadening by measuring a lumiflavin peak through the system with no column in place (see Supporting Information for details). Inset chromatogram shows measured separation (time axis is uncorrected for dead volume) of riboflavin and lumiflavin at 300 nL/min on a chip column packed with Raptor 5 µm superficially porous particles.

In Figure 3, each data point indicates the average *h* and *v* of three columns of the same particle and substrate type at a given flow rate, along with the standard deviation for these measurements. Based on these standard deviations, it is clear that column packing is more reproducible in the capillary format than it is for the microfluidic columns. When packing capillary columns there is a direct connection from the slurry to the outlet frit through the column blank, while the C-clamp fitting is also required when packing into the on-chip channel. The small dead volume that exists at this fitting interface could have an impact on the flow direction of particles going into the channel that is less reproducible column-to-column than a straight capillary channel. Additionally of note, the efficiency reproducibility decreases as the mobile phase velocity increases in all four column sets. This may be due to differences in packing quality affecting the slope of the *h*-*v* curve, which would magnify these differences at higher *v* values.

The worse performance of the microfluidic columns may be due to asymmetry of the channels (especially corner regions where significant differences in flow velocity can occur) compared to symmetrical capillaries [50–52]. Additionally, dead volumes in the C-clamp fitting and the sharp turn in flow at the beginning of the chip column as it transitions from the vertical to horizontal position (Figure 2C) may contribute to worse performance. In capillaries, these effects are eliminated. Because the packing method was identical for both formats (with pressure restricted to values limited by the C-clamp fitting), packing pressure is not a likely reason for the observed performance differences. Additionally, we were able to correct for the extra column band broadening due to the tubing from injector to chip (see the Supporting Information for details) so this is not a contributing factor. An interesting observation can also be found in Figure 3, where the slope of the *h*-*v* curve for the SPPs packed into the microfluidic channel is lower than that for the capillary columns (while it is the same for FPP columns in different substrates). Because it is believed that the extended wall region is a cause for increased *h*-*v* slopes of capillary-scale columns packed with SPPs due to transcolumn broadening [26], the asymmetric nature of the microfluidic channel could lead to disruptions in this wall effect and a decrease in slope magnitude.

Even with these issues, Figure 3 demonstrates both of these particle types can be used for high-speed LC even at low pressures while still maintaining reasonable column efficiency. For example, at *v* ≈ 10 with SPPs the average separation time was 54 s (corrected for injector to chip tubing), the average plate count was 2180, and the average pressure was 25 bar.

### 3.3. Column Permeability in Chips and Capillaries

**Figure 4:**
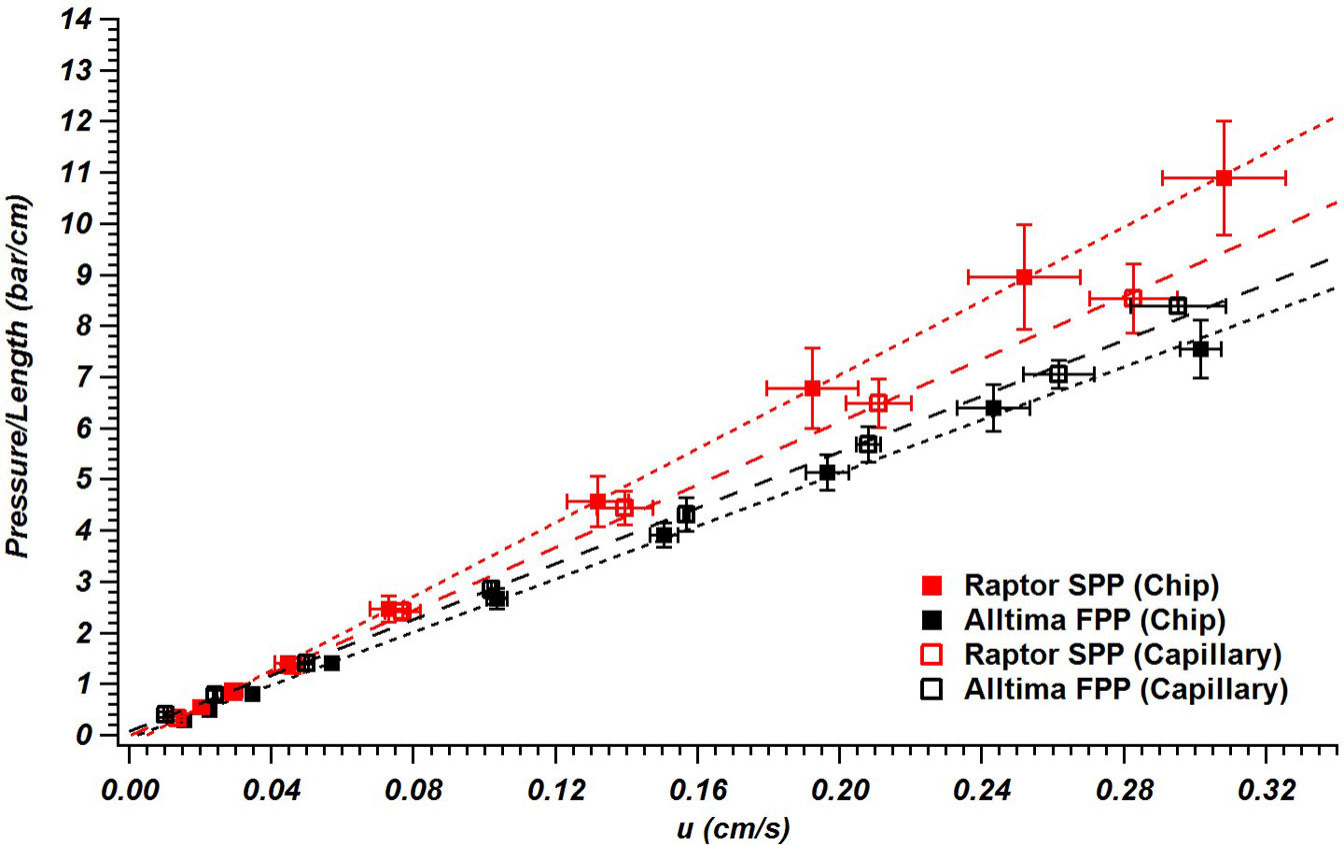
Pressure per unit length at varying mobile phase velocities for columns packed with Raptor 5 µm superficially porous particles and Alltima 5 µm fully porous particles into capillaries (column length ≈ 5.9 cm) and microfluidic chips (column length = 5.4 cm). Three columns of each type were packed and error bars reflect ±1 standard deviation (each data point corresponds to an equal pump flow rate).

Because one of the reasons SPPs were thought to be promising for microfluidic LC columns was their potential for low flow resistance at a given performance, we also compared pressure versus flow rate for the different columns (Figure 4). The SPPs allow reasonably fast flow at the pressure limits of the system For example, at the highest pressure tested, the flow rate was 600 nL/min allowing a corrected dead time of ~20 s.

The data also allow a rough comparison of similarly sized FPPs and SPPs. If the FPPs and SPPs were the same size and yielded the same interstitial porosity when packed, then the SPPs should have a higher permeability (when based on the linear velocity measured from an unretained dead time marker) because of their lower total porosity (due to the presence of the solid core) [19, 20]. Here, because the SPPs have a smaller diameter (see Table 1), an increase in flow resistance would be expected instead (since pressure is inversely related to the square of the particle diameter) [47]. In both column formats, this trend of higher flow resistance with the smaller particle diameters was observed (Figure 4). The magnitude of the difference varies between the substrates and is likely due to both variations in the particle structure [19], the channel geometry [50], and how different particle types interact with different wall shapes [24, 51]. While these properties have been previously studied for either particle type [24, 26] or channel geometry [50, 51], the combined effects are still not clear (especially for the newer SPP packing material used here) and will require further investigation.

For the FPPs, the column permeability in the chip was lower than in the capillary (Figure 4), which is probably due to the sharp corners only present in the microfluidic column where particles cannot pack as tightly [50–52]. This same trend was not observed in the SPPs, possibly due to differences in bed morphology and wall effects between different particle types [24, 26], as well as the impact of sharp corners being lower as particle size decreases.

## 4. Conclusions

The use of 4–5 µm SPPs packed into both capillaries and microfluidic channels was examined to determine their applicability for low-pressure LC of small molecules in miniaturized columns. The improvement in performance for SPPs compared to FPPs, especially with respect to *h_min_*, that have been demonstrated in larger column formats [17–20] was not observed for either capillaries or microchip columns, mirroring previous studies performed using particles less than 3 µm diameter [24–26, 28]; however, SPPs in chips did show flatter *h*-*v* response than FPPs in chips. Therefore, at the highest velocities tested, on-chip column performance approached that of the capillary columns, indicating they are a viable option for high-speed nano-LC. This trend may prove more advantageous for the separation of larger biomolecules where higher reduced velocities are achieved due to the lower diffusion coefficients. Additionally, better integration of injectors onto chips may improve performance.

## Supplementary Material

SI

## Figures and Tables

**Table 1:** Properties of prepared columns (and packing material) used in this study.

Particle	Structure	Size^a^	Capillary Efficiency^b^	Chip Efficiency^b^
Alltima C18	Fully Porous	4.97 ± 0.66 µm	110,000	41,400
Raptor C18	Superficially Porous	4.24 ± 0.16 µm	121,000	41,900

a As measured by SEM (averaged over 100 particles). Uncertainty to one standard deviation.

b Plates per meter calculated at *h_min,avg_*.
