# Supplementary material for: Evaluation of 5 µm Superficially Porous Particles for Capillary and Microfluidic LC Columns
Source: Chromatography (Basel). Author manuscript; Available in PMC 2015 Dec 3. (PMC4669065; doi:10.3390/chromatography2030502)
Supplement: SI [file NIHMS739031-supplement-SI.pdf]

Chromatography **2015**, *2*, 502-514; doi:10.3390/chromatography2030502

OPEN ACCESS

chromatography

ISSN 2227-9075

www.mdpi.com/journal/chromatography

Article

Evaluation of 5 µm Superficially Porous Particles for Capillary and Microfluidic LC Columns

James P. Grinias [1] and Robert T. Kennedy [1,2,*]

[1] Department of Chemistry, University of Michigan, Ann Arbor, MI 48109, USA; E-Mail: jgrinias@umich.edu

[2] Department of Pharmacology, University of Michigan, Ann Arbor, MI 48109, USA

* Author to whom correspondence should be addressed; E-Mail: rtkenn@umich.edu; Tel.: +1-734-615-4376.

Supplementary Materials

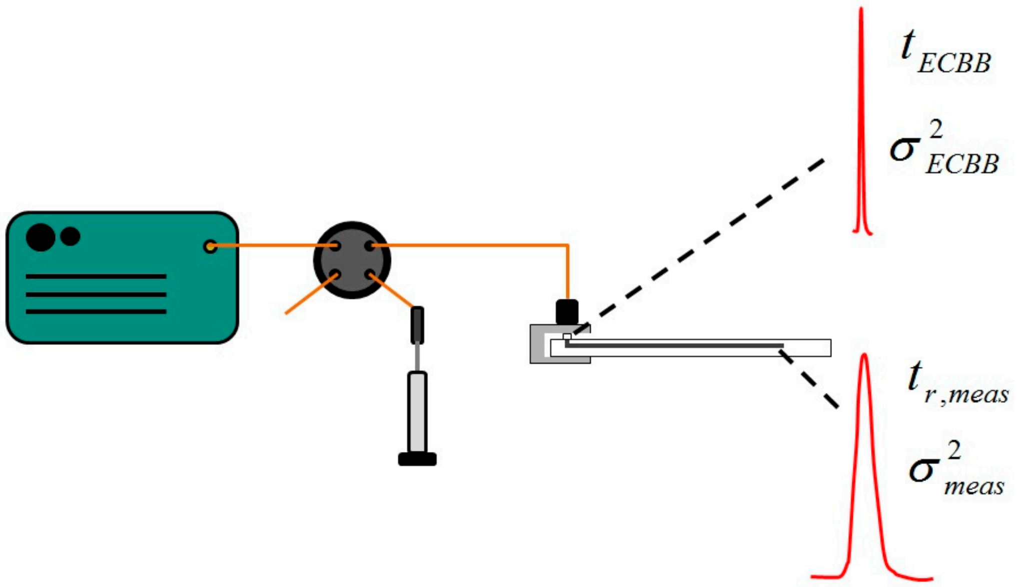

Figure S1. Diagram indicating measurements that must be made for extra-column band broadening corrections. The extra-column peak is measured at a detection window placed at the same point where the connecting tubing would reach the column inlet. This peak has an elution time t_{ECBB} and variance σ^2_{ECBB}. A second peak is then measured where the sample is injected, travels through the connecting tubing and the column, and is detected at the column outlet. This peak has an elution time $t_{r,meas}$ and variance σ^2_{meas}.

Methods for calculating chromatographic plate counts and mobile phase velocities [1] and correcting these calculations for extra-column band broadening [2] using the measurements described in Figure S2 have been detailed previously and will be discussed briefly here:

1. Retention times (t) and variances (σ^2) are measured with ($t_{r,meas}$ and σ^2_{meas}) and without (t_{ECBB} and σ^2_{ECBB}) the column in place.
2. The column retention time and column variances are calculated by subtracting these values:

$$t_{col} = t_{r,meas} - t_{ECBB} \tag{1}$$

$$\sigma^2_{col} = \sigma^2_{meas} - \sigma^2_{ECBB} \tag{2}$$

3. The column plate count (N) is determined by dividing the square of the retention time by the variance:

$$N_{col} = \frac{t^2_{col}}{\sigma^2_{col}} \tag{3}$$

4. Another measure of column efficiency, the plate height (H) of the column, is the number of plates in a given column length (from the inlet to the detection point):

$$H_{col} = \frac{L_{col}}{N_{col}} \tag{4}$$

5. The mobile phase velocity (u) is determined by the column elution time of a dead-time marker (riboflavin is used here) over that given column length:

$$u_{col} = \frac{t_{col,RiFl}}{L_{col}} \tag{5}$$

6. Reduced plate heights (h) and velocities (v) can then be calculated which account for differences in particle diameter (d_p), mobile phase, and/or analyte (of which the latter two can modify the diffusion coefficient (D_m)) when comparing different columns:

$$h = \frac{H_{col}}{d_p} \tag{6}$$

$$v = \frac{u_{col} \cdot d_p}{D_m} \tag{7}$$

Upon making these corrections, it was found that plate counts actually decreased by ~30% after making the corrections described above. In general, both t_{col} and σ^2_{col} were ~70% of their original measured value due to instrument effects. Based on Equation 3, this indicates that the calculated column plate count also drops to ~70% of the measured plate count (mainly since the dead time spent in the connecting tubing volume artificially increased the numerator). The effect was slightly greater in the columns packed with SPPs because the instrument volume was constant but the on-column volume was lower than FPPs due to the presence of the solid core, but still near the 30% loss generally observed. Although plate counts were actually lower than what was originally measured (prior to extra-column corrections), the mobile phase velocity ends up being about 40% higher based on the measured elution time for riboflavin (Equation 5). Thus, following data correction both plate heights and mobile phase

velocities end up being larger than expected (Equations 4 and 5), which shifts the *h*-*v* curves (Figure 3) up and to the right (Equations 6 and 7).

For the measured lumiflavin peak in this experiment, Raptor 5 µm superficially porous particles gave k' values near 0.2 and Alltima 5 µm fully porous particles had k' values near 0.3. Because there are fewer small molecules available that naturally fluoresce at 445 nm (the available wavelength for the LIF setup used in this experiment), mobile phase conditions were selected that enabled riboflavin to serve as a unretained dead time marker and lumiflavin to act as a retained analyte that could be measured to characterize column efficiency. However, because of the low k' values for lumiflavin, extra-column effects had more of an impact on measured efficiency than they would for a more retained analyte. In future experiments, other detection schemes can be implemented to measure different analytes with higher retention to reduce extra-column effects.

References

1. Neue, U.D. *HPLC Columns: Theory, Technology, and Practice*; Wiley-VCH: New York, NY, USA, 1997.
2. Gritti, F.; Felinger, A.; Guiochon, G. Influence of the errors made in the measurement of the extra-column volume on the accuracies of estimates of the column efficiency and the mass transfer kinetics parameters. *J. Chromatogr. A* **2006**, *1136*, 57–72.
